# Promoting Handwriting Fluency for Preschool and Elementary-Age Students: Meta-Analysis and Meta-Synthesis of Research From 2000 to 2020

**DOI:** 10.3389/fpsyg.2022.841573

**Published:** 2022-05-26

**Authors:** Carmen López-Escribano, Javier Martín-Babarro, Raquel Pérez-López

**Affiliations:** ^1^Research and Psychology in Education Department, Faculty of Education, Complutense University of Madrid, Madrid, Spain; ^2^Research and Psychology in Education Department, Faculty of Psychology, Complutense University of Madrid, Madrid, Spain

**Keywords:** handwriting, intervention, fluency, early writing, meta-analysis, systematic review

## Abstract

Handwriting is a complex activity that involves continuous interaction between lower-level handwriting and motor skills and higher-order cognitive processes. It is important to allocate mental resources to these high-order processes since these processes place a great demand on cognitive capacity. This is possible when lower-level skills such as transcription are effortlessness and fluent. Given that fluency is a value in virtually all areas of academic learning, schools should provide instructional activities to promote writing fluency from the first stages of learning to write. In an effort to determine if teaching handwriting enhances writing fluency, we conducted a systematic and meta-analytic review of the writing fluency intervention literature. We selected 31 studies: 21 true and quasi-experimental studies, 4 single-group design, 3 single-subject design, and 3 non-experimental studies, conducted with K-6 students in a regular school setting. A total of 2,030 students participated in these studies. When compared to no instruction or non-handwriting instructional conditions, teaching different handwriting intervention programs resulted in statistically significant greater writing fluency (ES = 0.64). Moreover, three specific handwriting interventions yielded statistically significant results in improving writing fluency, when compared to other handwriting interventions or to typical handwriting instruction conditions: handwriting focused on training timed transcription skills (ES = 0.49), multicomponent handwriting treatments (ES = 0.40), and performance feedback (ES = 0.36). There were not enough data to calculate the impact of sensory-motor and self-regulated strategy handwriting interventions on writing fluency. The significance of these findings for implementing and differentiating handwriting fluency instruction and guiding future research will be discussed.

## Introduction

Fluent and proficient writing communication abilities are crucial in our increasingly technical and literate society. While digital tools have become common, writing with paper and pen (or pencil) is the preferred tool for learning to write at schools, especially in the early grades (Santangelo and Graham, 2016), and is still considered a cultural technique not only for fulfilling academic requirements, but also in everyday life when writing a note or writing a shopping list (Wicki et al., 2014). In order to develop handwriting skills, children must begin building their foundation in kindergarten and elementary grades (Puranik et al., 2018).

Writing comprises several sequential and simultaneous language, cognitive, and motor processes, all of which demand some of the writer’s limited cognitive resources. Low level processes of handwriting involve an integration and coordination of spelling knowledge, allographic representations, and the execution of fine motor movements, while higher-level processes include planning, ideation, consideration of audience, and revising (Hurschler Lichtsteiner et al., 2018). Translating ideas into written language is not a significant problem for expert writers. Nonetheless, writing can be especially demanding for young children because their handwriting is not automated, and their motor processes are more capacity consuming than for adults (Peverly, 2006). Developing writers usually spend more time on lower-order processes than higher-order processes and may have limited knowledge of writing (Finlayson and McCrudden, 2019). Moreover, all the cognitive low- and high-level processes related to writing contribute to skilled writing, are interdependent and operate recursively with one another, they can interrupt each other and are embedded in each other (Berninger et al., 1996).

The most influential model of the cognitive processes on writing was proposed by Hayes and Flower (1980) and included three cognitive processes in skilled writing—planning, translating, and reviewing. In a subsequent revised version Hayes (2012) added other elements to the model like the task environment, the cognitive process of transcription, apart from planning, translation, evaluation, and motivation. Berninger et al. (1992) found that beginning writers had difficulty generating language to express ideas and lacked the knowledge of how to represent oral language orthographically. Thus, they added two components to the Hayes and Flower’s (1980) model: *text generation* and *transcription*. Accordingly, Berninger et al. (2002) presented the *Simple View of Writing* model that consists of three components: *transcription* (handwriting and spelling), *text generation* (translating generated ideas into written language), and *executive functions operations* (e.g., attention, planning, revising, and self-regulation) (Berninger et al., 2002, 2006). Berninger et al. (2002) stressed the key role of handwriting automation in their *Simple View of Writing* model, highlighting the importance of efficient and fluent execution of lower level processes in order to execute higher level metacognitive processes in composing a text. From a developmental point of view transcription and text generation dominate early writing, as the executive functions do not become prominent until students achieve self-regulation (Berninger et al., 2006). A subsequent study by Berninger et al. (2012) focused on the *transcription* component of writing and pointed out that in most instances the outcomes of this component could be text quality or production measures such as fluency or text length.

From the point of view of low- and high-level writing processes, the present study is focused on handwriting intervention to improve the automatization of allographic representations which is considered a low-level writing process. Considering, the above writing models this study is centered on the transcription component, specifically on how to teach handwriting to became fluent and automatic. In spite of the fact that the present research is centered in one element of writing, we understand that handwriting education needs to address all the elements of writing, be built on meaning-making and effective communication, and recognize social, linguistic, cognitive, affective, sensorimotor, motivational, and technological dimensions of writing development (Bazerman et al., 2017).

Berninger et al. (2006) emphasized fluency as an important aspect of writing development in the early grades to develop advanced text-generation skills. Fluency refers to automaticity and effortlessness in information processing (LaBerge and Samuels, 1974). According to information processing theory (LaBerge and Samuels, 1974), fluency is a developmental phenomenon, encompassing various grain sizes including sub-lexical, lexical, and text or discourse levels, and fluency at lower level is necessary for achieving fluency at a higher level (Wolf and Katzir-Cohen, 2001). Kim et al. (2018, p. 5) proposed “a developmental and componential definition of writing fluency as efficient and automatic writing connected texts, with accuracy, speed, and ease. In the beginning, text writing fluency is a function of transcription skills. With further development text writing fluency is efficiency and automaticity in writing text. When transcription is accurate, rapid, and effortless, cognitive resources such as attention and working memory can be allocated to meaning related processes, facilitating text generation.”

It takes students a long time to develop handwriting fluency at the expert level. In fact, the development of handwriting fluency continues to increase well beyond primary grades, at least until Grade 9 (Wicki et al., 2014; Alves and Limpo, 2015). Handwriting fluency and spelling significantly contribute to both writing quality and productivity (Kim et al., 2011). Moreover, automatic letter writing correlates with quantity and quality of written composition for both children (Graham et al., 1997; Jones and Christensen, 1999) and adult writers (Peverly, 2006). In addition, handwriting fluency continues to make a unique contribution beyond the primary grades in accounting for variability in how much and how well students write (Graham et al., 1997; Jones and Christensen, 2012). Unless automatic, the transcription processes can place so many demands on working memory that they interfere with other higher-order processes required for writing, such as planning and reviewing (Olive and Kellogg, 2002).

Findings of studies from different countries suggest that a very large percentage of students are experiencing difficulties with their writing skills (National Center for Education Statistics, 2012; Alves and Limpo, 2015; Koster et al., 2015). However, as Graham and Perin (2007) highlighted, not all the children that experience writing difficulties are identified as having a learning disability; low-achieving writers are included in this percentage of students with writing difficulties as a silent majority who lack writing proficiency but do not receive additional help. Moreover, a study by Puranik et al. (2018) reported that kindergarten teachers did not use any specific writing curriculum to teach writing, and most of the time consumed on writing instruction was spent on students writing independently. In a similar way, Vander Hart et al. (2010) study suggested that even though kindergarten teachers employ several effective intervention strategies for writing, there is room for improvement on implementing good handwriting practices based on research. In addition, studies by Gilbert and Graham (2010) and Koster et al. (2015) pointed out that the time devoted to writing in elementary schools is limited, and only a minority of schools and teachers used evidenced-based instructional practices.

Vander Hart et al. (2010) research on the in-depth analysis of handwriting curriculum in kindergarten classrooms found that writing fluency was a moderate priority in handwriting instruction. During the interviews, the teachers mentioned that their goal for their students was to learn how to form letters. They were not concerned about fluency and timed writing or writing from memory as these practices were never observed in kindergarten classrooms. Traditional handwriting lessons used to focus on legibility of handwriting and the importance and the knowhow of teaching handwriting fluency is not yet well known (Hurschler Lichtsteiner et al., 2018). A lack of opportunity to set up writing fluency skills in the elementary grades is particularly problematic because formal writing instruction is typically not offered to students after elementary school (Hier and Eckert, 2014). It seems likely that, without tailored supplementary support, these slow writers will be harshly constrained in their ability to enact high-level processes during text production (Limpo et al., 2018). Fluency-building interventions are conceptualized as time-efficient practices that can supplement instruction or intervention already occurring in the classroom (Martens et al., 2011).

Promoting writing fluency with evidence-based materials is particularly important in the initial years of learning to write. Nevertheless, previous reviews and meta-analyses on evidence-based writing interventions have focused mainly on children and youngsters in first grade and beyond; therefore, studies involving kindergartens were excluded and writing fluency-based outcomes were missing in most of the writing intervention reviews and meta-analyses done to date (Rogers and Graham, 2008; Graham and Sandmel, 2011; Graham et al., 2012, 2015; Koster et al., 2015; Graham and Harris, 2018). In this sense, it is worth mentioning Graham and Harris’s (2018) review, in which the authors conducted an extensive synthesis on writing instruction in elementary grades. Their research included 20 meta-analyses of true and quasi-experiments testing the effectiveness of one or more writing practices with children in grades from 1 to 12. The primary outcomes assessed by this study were writing quality, content learning, or reading comprehension. No writing fluency outcomes were included and none of the reviewed studies integrated instructional methods on how to teach handwriting skills to kindergarten children.

Nevertheless, Edwards (2003) review examined the literature on how to teach writing to kindergarten children. Writing and their instructional components related to handwriting, letter writing accuracy, spelling, fluency, and simple compositions are discussed in Edwards’ review, along with several handwriting instructional approaches, such as Alphabet Practice emphasizing letter formation and Alphabet Rockets targeting handwriting fluency. Besides, two meta-analyses provided findings on kindergarten children and on writing-based fluency interventions. Santangelo and Graham’s (2016) meta-analysis was conducted with students from kindergarten to 12th grade, they found that handwriting compared to no instruction or non-handwriting instructional conditions resulted in significantly greater legibility and fluency. Similarly, a previous work from the same authors (Santangelo and Graham, 2013) showed that handwriting instruction improved legibility and fluency in 18 studies including children from kindergarten to grade 7th.

Taking into consideration the Simple View of Writing model (Berninger et al., 2002), which emphasizes the idea that efficient or fluent execution of lower-level processes in writing development, and accounting for the scant research on handwriting fluency in the initial years of learning to write, the purpose of the present review is to identify instructional practices to increase writing fluency from kindergarten to 6th grade. In this line, a useful approach for identifying instructional practices that enhance the power to increase writing fluency is to conduct systematic reviews of writing fluency intervention research. Therefore, we intend to identify effective writing fluency instructional practices in kindergarten and elementary grade students by conducting a review of the writing fluency intervention literature.

It is important to identify writing fluency treatments with evidence of effectiveness, in order to provide teachers with instructional practices that potentially could improve the quality of their instruction and their students’ writing fluency. Moreover, applying evidence-based writing practices with students in earlier grades should reduce the number of youths who reach middle school not writing well enough to meet grade-level demands (Graham et al., 2012).

Although several meta-analyses and reviews targeting handwriting and strategy use have been published (Rogers and Graham, 2008; Graham and Sandmel, 2011; Hoy et al., 2011; Graham et al., 2012, 2015; Santangelo and Graham, 2013, 2016; Koster et al., 2015; Graham and Harris, 2018), there has not been a comprehensive systematic review targeting writing fluency interventions that was conducted focused on kindergarten and elementary grade students.

The present paper reports a meta-analysis of handwriting fluency intervention research involving true and quasi-experimental and single-group design studies. It also includes a review of single subject design and non-experimental studies to draw a broad set of recommendations for teaching handwriting fluency-based interventions, applying the principle to make the best of the available data, not simply experimental effect sizes (Pressley et al., 2006). It focused broadly on teaching handwriting fluency to K-6 students in regular school settings (i.e., not schools exclusively for students with special needs). Therefore, the final goal was to systematically review the effectiveness of the impact on writing fluency of handwriting interventions to both update and better quantify the conclusions from a narrative review and meta-analysis perspective. Concretely, the objectives of this review were: (1) to perform a systematic review and meta-analysis about the effectiveness of handwriting instruction compared to conditions involving either no instruction or instruction unrelated to handwriting and (2) to determine the effectiveness of specific handwriting programs (e.g., handwriting based on transcription skills, combined handwriting treatments, multicomponent handwriting interventions, sensory-motor handwriting treatment, self-regulated strategy, and performance feedback) used to improve writing fluency in the pretest and posttest outcomes compared to other handwriting conditions. If an intervention category had at least three studies that reported outcomes for a congruent measure of writing fluency, we performed a meta-analysis; otherwise, we reviewed the studies and presented a conclusion.

## Method

### Inclusion and Exclusion Criteria

The selection of the articles was made taking into consideration the following inclusion criteria: (a) interventions and theoretical approaches to improve writing fluency; (b) students aged between 5 and 12 years; (c) students enrolled in general education classrooms; (d) not experiencing severe motor and/or perceptual deficits that precluded students from handwriting; (e) not experiencing significant cognitive and/or developmental deficits; (f) not second language acquisition (SLA) students; (g) articles published in scientific journals between 2000 and 2020; (h) writing intervention done on paper. Research with the main focus on the assessment of writing fluency was rejected.

### Location and Selection of Studies

A pairwise search of articles published in English between 2000 and 2020 was carried out in the following databases: WoS, ERIC, Scopus, PROQUEST, Medline, and PubMed. To search papers related to the present study topic, we inserted a combination of the following keywords: *writing OR handwriting AND fluency OR automaticity OR speed AND intervention OR instruction OR training OR treatment OR teaching*, considering only the category of journal articles.

A total of 1,120 publications were found, 318 from WoS database, 210 from Scopus, 235 from PROQUEST, 211 from Medline and 146 from PubMed. Additionally, eight records were identified through other sources. All of them were exported to the Mendeley program for further sorting and selection. Subsequently, duplicate articles were eliminated and a total of 747 publications were obtained to be analyzed. Next, the three authors made the independent selection of each study and resolved disagreements. Once the inclusion and exclusion criteria had been analyzed and applied, a total of 31 articles were obtained. All of them were coded and included in Table 1.

**TABLE 1:** Summary of reviewed studies categorized by intervention type.

Study first author (year)	Type of study	Description of conditions and (n) in each condition	Grade	Student type	Sessions	Quality score	Flu. ES*^a^*
**Handwriting-Transcription/True-QES studies**							
Alves et al., 2016 *^b,c^*	True-QES	1. Handwriting (*n* = 18) 2. Spelling (*n* = 17) 3. Keyboarding (*n* = 20)	Second grade	Full range	10 weeks units	9	0.82
Graham et al., 2000 *^b,c^*	True-QES	1. Handwriting (*n* = 15) 2. Phonological awareness (*n* = 15)	First grade	Struggling writers and learning disabilities	27 sessions	9	0.77
Howe et al., 2013 *^c^*	True-QES	1. Intensive Practice from the handwriting curriculum (*n* = 34) 2. Visual-Perceptual-Motor skills (*n* = 38)	First and second grades	Full range and struggling writers	12 weeks	6	-0.05
**Handwriting-Transcription/Single group design study**							
Mackay et al., 2010	Single group design	1. Log Handwriting Program (LHP) (*N* = 16)	First and second grades	Struggling writers	8 weeks	7	-0.49
**Handwriting-Transcription/Single subject design study**							
Limpo et al., 2018	Single subject design	1. Handwriting intervention (N = 3)	Fifth grade	Struggling writers	5 weeks	6	2.14
**Combined Handwriting Instruction/True-QES studies**							
Graham et al., 2018 *^b^*	True-QES	1. Handwriting + spelling (*n* = 15) 2. Phonological awareness (*n* = 15)	First grade	Learning disabilities	8 sessions	9	0.95
Hurschler Lichtsteiner et al., 2018	True - QES	1. Handwriting + spelling (*n* = 78) 2. Spelling (*n* = 36)y 3. Handwriting (*n* = 34) 4. Reading (*n* = 27)	Third grade	Full range	5 weeks	6.5	–
Limpo and Alves, 2018 *^b^*	True - QES	1. Transcription + self-regulation (*n* = 43) 2. Self-regulation (*n* = 37) 3. Typical handwriting instruction (*n* = 39)	Second grade	Full range	10 sessions	7	0.58
**Multicomponent programs/True-QES studies**							
Case-Smith et al., 2014b *^b,c^*	True-QES	1. Write Star Program (*n* = 37) 2. Typical handwriting instruction (*n* = 30)	First grade	Full range	12 weeks	9	0.46
Case-Smith et al., 2014b *^b,c^*	True-QES	1. Write Star Program (*n* = 77) 2. Typical handwriting instruction (*n* = 55)	First grade	Full range	12 weeks	9	0.06
Puranik et al., 2017 *^b,c^*	True-QES	1. Peer Assisted Writing Instruction (PAWS) (*n* = 22) 2. Typical handwriting instruction (*n* = 62)	KG	Full range	35 sessions	9	0.55
Puranik et al., 2018 *^b,c^*	True-QES	1. PAWS (*n* = 78) 2. Typical handwriting instruction (*n* = 71)	KG	Full range	26 weeks	10	0.69
Van Waelvelde et al., 2017 *^b,c^*	True-QES	1. I can! Program (*n* = 18) 2. Delayed instruction (*n* = 13)	7 and 8 years old	Struggling writers	7 weeks	8	0.27
**Multicomponent programs/Single group design studies**							
Case-Smith et al., 2011	Single group design	1. Write Star Program (*N* = 17)	First grade	Full range	12 weeks	7.5	1.61
Case-Smith et al., 2012	Single group design	1. Write Star Program (*N* = 36)	First grade	Full range	12 weeks	7	0.87
**Multicomponent programs/Single subject design studies**							
Hansen and Wills, 2014	Single subject design	1. Handwriting + goal setting + contingent reward (*N* = 1)	Ten-year-old student	Struggling writers	20 sessions	4.5	–
**Sensory-motor handwriting/True-QES studies**							
Bara and Bonneton-Botté, 2017	True-QES	1. Whole body visuo-motor (*n* = 36) 2. Visual teaching (*n* = 36)	KG	Full range	6 weeks	6	–
Salls et al., 2013	True-QES	1. Handwriting Without Tears (*n* = 14) 2. Peterson directed program (*n* = 17)	First grade	Full range	One school year	7	0.16
Study first author (year)	Type of study	Description of conditions and (n) in each condition	Grade	Student type	Sessions	Quality score	Effect size
Weintraub et al., 2009 *^b^*	True-QES	1. Sensorio-motor (*n* = 19) 2. Task oriented interventions (*n* = 13) 3. Typical handwriting instruction (*n* = 17)	Second, third, and fourth grades	Struggling writers	8 sessions	7	0.07
**Sensory-motor handwriting/Single group design study**							
Roberts et al., 2010	Single group design	1. Kinesthetic cursive handwriting program (*n* = 28)	Fourth, fifth and sixth grades	Struggling writers	7 weeks	6	0.86
**Self-regulated strategy/True-QES study**							
Jongmans et al., 2003	True-QES	1. Handwriting self-instruction (*n* = 7) 2. Typical handwriting instruction (*n* = 7)	7.92 years old	Struggling writers	18 sessions	6	-0.28
**Self-regulated strategy/Single subject design study**							
Geisler et al., 2009	Single subject design	1. Self-counting + A synonym list (*N* = 5)	First grade	High ability	25 sessions	5	–
**Self-regulated strategy/Single subject Non experimental studies**							
Kasper-Ferguson and Moxley, 2002	Non-exp.	1. Students counting and graphing words + sample writing (*N* = 20)	Fourth grade	Full range	One school year	4	–
Zumbrunn and Bruning, 2012	Non-exp.	1. Self-regulated strategy development (*N* = 6)	First grade	Full range	Spring term	6	–
**Performance feedback/True-QES studies**							
Alitto et al., 2016 *^c^*	True-QES	CBM-WE 1. Performance feedback and goal setting (*n* = 57) 2. Practice only (*n* = 57)	Fourth and fifth grades	Full range and learning disabilities	10 weeks	10	0.26
Hier and Eckert, 2014 *^c^*	True-QES	CBM-WE 1. Performance feedback (*n* = 51) 2. Practice only (*n* = 52)	Third grade	Full range	12 weeks	9	0.54
Hier and Eckert, 2016 *^c^*	True-QES	CBM-WE 1. Performance feedback (*n* = 34) 2. Practice only (*n* = 33) 3. Multiexemplar training (*n* = 41)	Third grade	Full range	9 weeks	8	0.05
Study first author (year)	Type of study	Description of conditions and (n) in each condition	Grade	Student type	Sessions	Quality score	Effect size
Koenig et al., 2016 *^b,c^*	True-QES	CBM-WE 1. Performance feedback + goal setting (*n* = 39) 2. Performance feedback (*n* = 39) 3. Typical handwriting instruction (*n* = 39)	Third grade	Full range	8 weeks	10	-0.18
Roth and Guinee, 2011 *^b,c^*	True-QES	1. Interactive writing + teacher feedback (*n* = 49) 2. Typical handwriting instruction (*n* = 52)	First grade	Full range	One school year	8	0.81
Truckenmiller et al., 2014 *^b,c^*	True-QES	CBM-WE 1. Performance feedback (*n* = 46) 2. Typical handwriting instruction (*n* = 48) 3. Practice only (*n* = 39)	Third grade	Full range and learning disabilities	8 weeks	9	0.66
**Performance feedback/Non-exp. study**							
Heskial and Wamba, 2013	Non-exp.	1. Collaborative inquiry/action with teacher feedback (*N* = 120)	KG	3 full range classes and 1 learning disabilities	Daily 50 min writing work-shops	3.5	–

*^a^When ES could be calculated between conditions 1 and 2, the result is shown; otherwise, the studies that did not allow the calculation of the effect size are marked with a line.*

*^b^Study included in the analysis of the first objective.*

*^c^Studies include in the analysis of the second objective.*

*True-QES, True quasiexperimental; KG, kindergarten; Non-Exp, non-experimental. Studies are organized by intervention category and alphabetical order; fluency effect sizes were calculated between condition 1 and condition 2.*

*Studies marked with this symbol were selected for calculating the effectiveness of handwriting instruction compared to conditions involving no instruction or instruction unrelated to handwriting.

**FIGURE 1:**
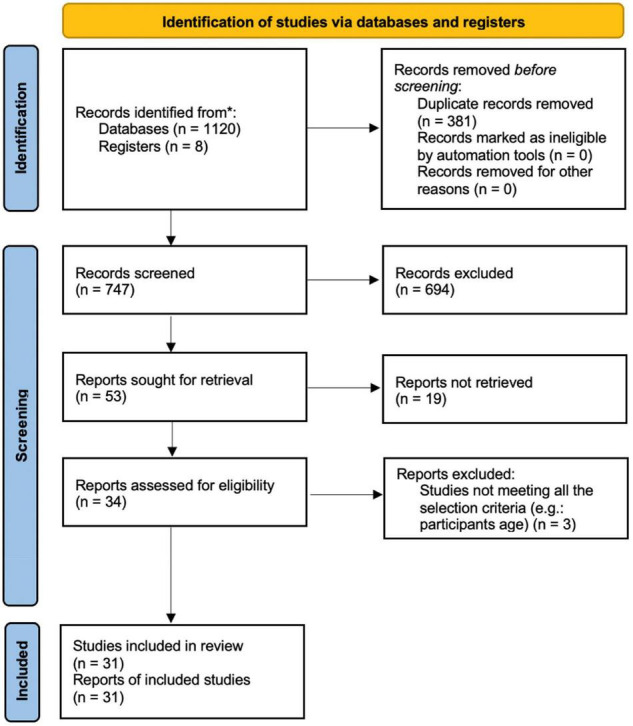
PRISMA flow diagram (Page et al., 2021).

This study followed guidelines proposed by Preferred Reporting Method for Systematic Reviews (PRISMA Declaration), according to the flowchart of four phases (Figure 1) in addition to its checklist and report items (Page et al., 2021).

### Categorizing Interventions

For the analysis, the first author examined each study and grouped them according to their main focus of intervention. Next, a list of handwriting intervention categories was developed collaboratively by the authors. Once the list was created it was reread again by the authors and the categories were refined and each study placed in its respective category. Studies with a comparable focus of intervention were grouped into categories, based on those used in previous meta-analyses (e.g., Graham and Perin, 2007; Santangelo and Graham, 2016; Koster et al., 2015).

At least one or more studies examined the effectiveness of the following categories (1) handwriting/transcription, (2) sensory-motor handwriting, (3) spelling, (4) peer-assisted learning, (5) self-regulated strategy, (6) performance feedback, (7) goal setting, and (8) contingent reward. Table 2 presents a definition of the eight treatment categories found in the reviewed studies.

**TABLE 2:** Identification of writing fluency treatments.

Treatments	Description	Authors
Handwriting/transcription	Handwriting treatment consists of explicit instruction and intensive practice in writing letters, words, and sentences to promote legibility, handwriting speed, writing fluency, and writing expression. The most common handwriting instruction tasks are writing and modeling letter formation, fostering automatic letter production and copying exercises	Graham et al., 2000; Howe et al., 2013; Alves et al., 2016
Sensory-motor handwriting	Sensory-motor handwriting practices include visual, auditory, tactile, rhythm, and movement techniques to reinforce letter formation, size, and alignment. Usually in sensory-motor handwriting practices, letters are taught in groups that share movement patterns	Weintraub et al., 2009; Salls et al., 2013; Hansen and Wills, 2014; Bara and Bonneton-Botté, 2017
Spelling	In spelling treatments, students (depending on the characteristics of the language) receive explicit instruction and practice in the alphabetic principle and its alternations, vowel sounds, onset and rime, and morphemic structures of words, as well as spelling patterns. Spelling lessons usually follow a sequence of increasing complexity from consistent or rule-based spelling patterns to inconsistent alternations and complex spelling patterns	Graham et al., 2018; Hurschler Lichtsteiner et al., 2018
Peer-assisted learning strategy	Peer-assisted learning strategies involve peers helping one another to write, to practice, and to learn themselves by teaching others on how to write. Commenting on a peer’s work can make students aware of their own writing and help children build metacognitive/metalinguistic skills	Puranik et al., 2017; Puranik et al., 2018
Self-regulated strategy	Self-regulated writing consists of teaching children specific strategies for planning and writing a complete story: to set goals, to monitor their understanding of the writing process, and to evaluate the written text. Consequently, some self-regulated strategies regulate performance (e.g., self-instruction), the observation of one’s progress (e.g., self-monitoring), and the evaluation of the written task (e.g., self-evaluation)	Jongmans et al., 2003; Limpo and Alves, 2018
Performance feedback	Performance feedback is a means by which students receive objective information on their task mastery. Performance feedback could be presented in both visual and oral formats. The visual presentation could be, for example, in the form of a page that includes numeric feedback and a graphic or an arrow pointing up or down, indicating whether performance increased or decreased. The oral presentation could be completed by the teacher who reviewed the information presented on the feedback page	Hier and Eckert, 2014; Truckenmiller et al., 2014; Alitto et al., 2016; Hier and Eckert, 2016; Koenig et al., 2016
Goal setting	Goal setting involves the design of an action plan aimed to motivate and guide a student toward a defined goal or a set of goals. Goals must be provided in a clear, objective way that is easily understood by the individual receiving the intervention	Alitto et al., 2016; Koenig et al., 2016
Contingent reward	Contingent rewards consist of a motivational-based system that is used to reward students that meet their identified goals by providing reinforcement for a job well done	Hansen and Wills, 2014

The goal of this categorization process was to create groups of studies that isolated specific teaching methods. There were three exceptions to this basic approach (per the previously described questions of this review). We created one category that compared any handwriting intervention to a control condition that did not involve a handwriting treatment. In addition, two more categories were added for studies that fit in more than one category previously described: combined handwriting instruction and multicomponent programs. Studies in the combined handwriting instruction included investigations where handwriting was combined with other practices, such as handwriting and spelling (e.g., Graham et al., 2018). Multicomponent interventions included skill-based combined with performance-based writing treatments (e.g., Case-Smith et al., 2011; Puranik et al., 2017).

In addition, it should be noted that a study could be placed in more than one category, as some studies included more than one intervention. For example, Koenig et al. (2016) included performance feedback and goal setting as well as a non-intervention group. Since performance feedback was the experimental category, it was included in the performance feedback category.

### Coding and Data Analysis

First, two independent investigators coded all the studies considering main different aspects: Grade, Participants, Type of Study, Writing intervention, and Measurements. Percentage of agreement was: 90.3, 93.5, 80.6, 90.3, 93.5, and 71.0%, respectively. Subsequently, several meetings were held to debate and share the information included in the finally selected articles. The coding sheet collected information on the following aspects (see Table 1):

• Study first author (year).
• Type of study: Indicates the category of the research. true or quasi-experimental; single-group design; single-subject design; non-experimental or qualitative studies.
• Description of conditions and (n): Presents a list of the study conditions and the participants in each condition. Studies are categorized by types of intervention and alphabetical order.
• Grade: Applies to participants’ grade level; when the study did not mention age, this was reported.
• Student type: As Santangelo and Graham. (2016) stated, there were three categories for the type of participant: (1) full range for students with typical handwriting skills; (2) high for those with above-average handwriting skills; (3) struggling for students with significant handwriting difficulties. When none of the previous conditions was specified, full range was reported. We added the category of learning disabilities when the authors explicitly mentioned it.
• Sessions: The length of the intervention in weeks or sessions, as defined by the authors. In a few cases, the only data available was the number of semesters or the academic year.
• Quality score: This was calculated considering the quality indicators of Gersten et al. (2005). These indicators refer to: (a) adequate information about participants; (b) methods to guarantee participants had been randomly assigned; (c) appropriate explanation regarding interventionists or teachers running the intervention; (d) sufficient details of the intervention, its description and assessment; (e) treatment fidelity; (f) report of the characteristics and evaluation of comparison conditions; (g) variety of measures to assess all the variables; (h) results that prove the impact of the intervention; (i) adequate data analysis techniques; and (j) presence of inferential statistics and effect size estimations. When the indicator was met 1 point was assigned, and when it was not, no points were assigned; therefore, the maximum score was 10. Nevertheless, and since some of the indicators specified two criteria, 0.5 points were given, for example in the indicator (d) some researchers might describe the intervention but not its evaluation.
• Flu. ES: This calculation is explained below (section “Results”).

Table 3 presents the findings from analysis of 10 quality indicators (described in Table 4). Across all the selected studies the mean quality score was 8.5 (SD = 1.13), for the *Handwriting interventions studies* 8.3 (SD = 1.15), for *Handwriting instruction studies* 8.8 (1.10), for *Multicomponent handwriting treatments studies*, and 9.0 (1.14) for *Performance feedback studies*. Therefore, most of the chosen studies included in the performed meta-analyses according to the two objectives of the study were of significant quality. Considering the type of studies, *Handwriting instruction studies* got the highest adherence (100%) to most of the quality indicators, even though none of them report information regarding the interventionists or teachers conducting the program. On the contrary, *Handwriting instruction studies* were found as the lowest adherence research scoring the lowest value for 0% in the Interventionists or teachers’ information indicator. Taking all type of studies, the quality indicators with most adherence (75.1%) were Participant’s description, Intervention description, Comparison conditions reported, and Multiple measures.

**TABLE 3:** Total quality score and percentage of studies in which a quality indicator by types of study.

	Total score*	Quality Indicators									
Type of Study	*M (SD)*	*PT* (%)	*MR* (%)	*ITI* (%)	*ID* (%)	*TF* (%)	*CCR* (%)	*MM* (%)	*RPII* (%)	*DAT* (%)	*PIS* (%)
Handwriting interventions compared to conditions with no instruction or unrelated to handwriting studies (*k* = 13)	8.5 (1.13)	92.3	69.2	38.5	92.3	76.9	92.3	92.3	84.6	69.2	69.2
Handwriting instruction studies (*k* = 3)	8.3 (1.15)	100.0	100.0	0.0	100.0	66.7	100.0	100.0	100.0	100.0	66.7
Multicomponent handwriting treatments studies (*k* = 5)	8.8 (1.10)	100.0	60.0	80.0	100.0	80.0	100.0	100.0	100.0	80.0	80.0
Performance feedback studies (*k* = 6)	9.0 (1.14)	100.0	83.3	66.7	100.0	100.0	100.0	100.0	83.3	83.3	83.3
TOTAL (*k* = 17)	8.6 (1.06)	75.1	59.2	33.7	75.1	61.4	75.1	75.1	73.6	63.2	59.8

k number of studies; M mean; SD. *Total quality score is the sum of all quality indicators (scale 0–10).

*PT, participant’s description; MR, methods randomization; ITI, interventionists or teachers’ information; ID, intervention description; TF, treatment fidelity; CCR, comparison.*

**TABLE 4:** Definitions for quality indicators.

Quality Indicator	Definition
*Participant’s description*	Provides sufficient information about participants to be informed about whether they present learning difficulties
*Methods randomization*	Follows adequate procedures for randomly assigning participants across conditions
*Interventionists or teachers’ information*	Properly describes information related to those who conduct the interventions (either professionals or teachers) such as years of experience
*Intervention description*	Reports the type of intervention/programs used in the research, specifying instructions, materials, sessions, etc.
*Treatment fidelity*	Explains the extent to which the intervention has been implemented as planned
*Comparison conditions reported*	Includes the description and document the treatment implementation in the comparison group along with its assessment
*Multiple measures*	Incorporates a variety of valid and consistent measurements that line up with the objectives of the intervention
*Results proving intervention’s impact*	Reports all the results showing the impact of the intervention at the appropriate times (immediately, long-term effects, etc.)
*Data analysis techniques*	The selected analysis of the data fits the research questions and hypotheses established by the authors
*Presence of inferential statistics*	Reports inferential statistics and effect sizes estimations

Second, when data were available for true and quasi-experimental studies (True-QES) and for single-group design studies, Cohen’s d values with pooled 95% CIs were used to estimate the effect size (ES) of each study (see Table 1). Before calculating ES, for True-QES, an adjustment was made to the scores of the comparison groups. Means for each pretest and posttest group were subtracted separately, then the ES was calculated by subtracting the adjusted posttest scores of one group from the adjusted posttest score of the other comparison group and dividing it by the pooled standard deviation. Positive Cohen’s d values indicated a greater gain in the intervention group versus the control group or versus other intervention groups, while a negative value indicated the opposite, i.e., a greater gain in the control group or in groups with other types of intervention.

Then, in the case of the single-group design studies, the positive values of Cohen’s d indicated an increase between pre-treatment and post-treatment in the variable under study. When the Cohen’s d value was negative, the value of the pre-treatment measure was greater than the value of the post-treatment measure and therefore there had been no gain associated with the application of the program over time.

After calculating each intervention ES of True-QES, following our first research goal, we ran a meta-analysis considering the studies where the experimental group or intervention under study was compared to control groups or to other groups with an intervention not related to writing. Studies under consideration to calculate this meta-analysis are marked with a (***^b^***) in Table 1. Fifteen studies were finally included. The results of the meta-analysis are shown in Table 5.

**TABLE 5:** Summary of results for each research question.

	k	ES	95% CI	Q	I^2^
Objective 1. Handwriting interventions compared to conditions involving either no instruction or instruction unrelated to handwriting?	13 True-QES	0.66	0.51 to 0.81	10.83	0
Objective 2: Effectiveness of specific methods and strategies used to improve handwriting fluency*^a^*					
Handwriting instruction	3 True-QES	0.49	–0.10 to 0.99	4.64	56.87
Multicomponent handwriting treatments	5 True-QES	0.51	0.38 to 0.63	1.60	0
Performance feedback	6 True-QES	0.36	0.06 to 0.66	16.86**	70.34

*^a^Combined handwriting treatments, sensory-motor handwriting treatment and self-regulated strategies did not meet the criteria of a minimum of three investigations to perform a meta-analysis calculation.*

*True-QES, true quasiexperiments on writing fluency; k, number of studies; ES, effect size; CI, confidence interval.*

*p < 0.05.

**p < 0.01.

Another meta-analysis was conducted in accordance with our second study goal. Following a previous meta-analysis (Santangelo and Graham, 2016), if an intervention category had at least three studies that reported outcomes for a congruent measure of fluency, we computed an average weighted ES; otherwise, we reviewed the studies and presented a conclusion. Only three handwriting intervention categories met this condition: handwriting/transcription instruction, multicomponent handwriting treatments, and performance feedback (see Table 5).

Meta-Essential software was used to perform four meta-analyses (Suurmond et al., 2017). We followed the Hedges (1981) procedure in order to correct bias due to the small sample sizes. As Cohen (1988) proposed, the magnitude of the ES was interpreted from small to large (d = 0.2 small, d = 0.5 medium, d = 0.8 large). Statistical heterogeneity of ES values was analyzed by applying several methods: The Q statistic and the I^2^ index (Cooper et al., 2019), by visually inspecting funnel plots (Borenstein et al., 2009) and the linear regression test proposed by Egger et al. (1997). Using the guidelines proposed by Higgins and Green (2011), we interpreted heterogeneity, i.e., 0–40% might not be important, 30–60% may represent moderate heterogeneity, 50–90% may represent substantial heterogeneity, and 75–100% may imply considerable heterogeneity.

To carry out both meta-analyses with regard to the similarity of outcome measures, ES were only calculated when the research measured the same construct: writing fluency. It should be noted that we considered all those measures related to writing fluency, even though researchers assessed the same construct in different ways. The outcomes measures used were based on comparable assessments (e.g., number of words and sentences spelled correctly in 3 min based on a curriculum-based measurement in written expression (CBM-WE) probes, a copying task in 5 min, letters per minute, and other measures and test of writing fluency such as the rate scale of the Minnesota Handwriting Assessment by Reisman (1999). When a study had several measures that could be considered as writing fluency or several studies, a single measure was obtained by averaging the individual effects.

Finally, due to the small number of participants, we did not include the analysis of single-subject design from Table 1. Nevertheless, we considered including single-subject design studies and qualitative or non-experimental studies in order to identify the greatest amount of relevant evidence on the subject under analysis. The aim was to classify, compare, translate, display, and analyze the information included in these studies in order to make further interpretations (Jensen and Allen, 1996). We read, identified, and coded the practices on writing fluency for each single-subject and non-experimental study.

## Results

The results of our meta-analysis are summarized in Table 1, reporting the ES for each study, and Table 5 reporting the number of studies used in the analysis, combined and simple ES, levels of statistical significance, CIs, and measures of homogeneity, i.e., Q and I^2^.

### First Study Objective: Effectiveness of Any Type of Handwriting Intervention Compared to Conditions Involving Either No Instruction or Instruction Unrelated to Handwriting

We aimed to search whether or not the effects of any type of handwriting instruction produce greater gains in writing fluency than non-handwriting instruction or instruction non-related to handwriting. A total of 13 True-QES investigations were analyzed to examine whether any type of handwriting-based interventions improves writing fluency versus non-intervention or non-handwriting-related instruction. From the total of studies analyzed all showed positive results. In total, these studies comprised a total of 1,111 students and a range of sessions between five weeks and the whole school year.

**FIGURE 2:**
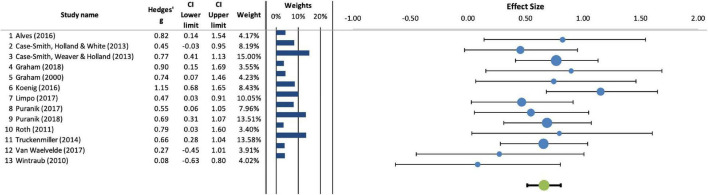
Effectiveness of handwriting interventions compared to conditions involving either no instruction or instruction unrelated to handwriting.

The results show a combined ES of 0.66 [95% CI (0.51, 0.81)]. Regarding the heterogeneity statistic, the Q statistic was not significant and the I^2^ statistic indicated that 0% of the variance was produced by variations between the studies. The fail-safe N showed that 148 studies would be necessary to make the combined ES statistically insignificant. With the trim and fill method, no studies were imputed in the funnel plot to alleviate difficulties with missing studies showing negative effects. Figure 2 shows the effectiveness of the different programs used with respect to no instruction or instruction unrelated to handwriting.

### Second Study Objective: Effectiveness of Specific HandWriting Methods and Strategies to Improve Writing Fluency Compared to Other Handwriting Conditions

The main aim was to analyze whether any type of handwriting intervention improves handwriting compared to other conditions involving handwriting fluency interventions or no instruction. In any case, the calculated ES to perform these meta-analyses for a specific handwriting method was the result of comparing condition 1 to condition 2 (see Table 1).

#### Handwriting/Transcription

We found five studies that used handwriting instruction, and included the participation of 176 students. The interventions in these handwriting investigations ranged from short 5-week treatments to longer interventions with 27 sessions. Although the intervention conditions all involved handwriting instruction, the specific tasks of focus varied across studies (e.g., writing letters, words and sentences fluently and accurately, copying exercises, writing letters with correct alignment, size and spacing). Three of the studies used a true or quasi-experimental design; the first two of them showed a gain in the treatment group or condition 1 compared to condition 2 (Graham et al., 2000; Alves et al., 2016). However, the third study did not show effectiveness of treatment (Howe et al., 2013). Together, they showed a combined ES of 0.49 [95% CI (–0.10, 0.99)]. The Q statistic was not significant and the I^2^ statistic indicated that 56.87% of the variance was explained by variability between the studies, indicating moderate heterogeneity. The Fail-safe N indicated that 793 missing studies would be needed to reverse this finding. The control conditions of these two studies were quite diverse (see Table 1). Two studies used a single group design. The first (Mackay et al., 2010) indicated a decrease in writing fluency (ES = –0.49) and the second study (Limpo et al., 2018) was excluded from the overall ES analysis of this question due to the small sample size. Despite this, the last study revealed that handwriting intervention through fast-paced alphabet and copying activities was highly effective in increasing students’ handwriting fluency, as the calculated ES shows (see Table 1).

#### Combined Handwriting

In the following question, it was analyzed whether combined treatments on handwriting were effective. These studies analyzed a total of 324 students and comprised between 5 and 10 treatment sessions. Of the studies analyzed, only three showed combined treatments (Graham et al., 2018; Hurschler Lichtsteiner et al., 2018; Limpo and Alves, 2018). Two of them had a true or quasi-experimental design showing positive results (Table 1). The study carried out by Hurschler Lichtsteiner et al. (2018), did not allow for a calculation of ES; however, the study results reported that as handwriting automaticity was high at the beginning of the study, the intervention was not able to improve it further.

#### Multicomponent Handwriting

A total of eight studies analyzed whether students who received multicomponent treatment in writing fluency showed improvements in the mentioned variables compared to those students who did not receive it. These studies comprised the participation of 464 students and the duration of treatment varied between 12 and 35 sessions. From the eight studies, five were true or quasi-experimental (Case-Smith et al., 2014a,b; Puranik et al., 2017, 2018; Van Waelvelde et al., 2017) and showed a combined ES of 0.51 [95% CI (0.38, 0.63)]. The Q statistic was not significant and the I^2^ statistic with a value of 0 indicated that the effects were homogeneous. Two other studies, Case-Smith et al. (2011) and Case-Smith et al. (2012), had a single group design and indicated a positive gain in the treatment group with an ES of 1.61 and 0.87, respectively. In addition, the study by Hansen and Wills (2014) described in a case study a male student in elementary school who, after the performance-based intervention (goal setting and contingent reward) and skill-based intervention (handwriting instruction based on writing complete sentences), increased the number of complete sentences written.

#### Sensory-Motor Handwriting

We found four studies, all comparing the impact of sensory-motor handwriting treatment on writing fluency. In total, 225 students participated in these studies and the amount of instruction provided ranged from six weeks to one year. Two of the studies had a true or quasi-experimental design and they showed no effect of intervention or it was very low (Weintraub et al., 2009; Salls et al., 2013). A third study, conducted by Bara and Bonneton-Botté (2017), assessed the impact of a teacher-implemented visuomotor intervention program. They compared a visuomotor program in which letters were explored with the arm and whole body, with a typical visual training program. This study was not included in the analysis because results on writing fluency were not clearly stated and were somehow contradictory. Finally, the study by Roberts et al. (2010) with a single group design investigated whether students participating in a kinesthetic writing program intervention improved speed. Although the calculations showed a positive effect of the program, the authors claimed that the increase in handwriting speed was not clinically significant.

#### Self-Regulated Strategies

Regarding this category, four studies were analyzed to test whether or not students who received self-regulation strategies showed gains in writing fluency compared to those students who did not receive this kind of technique or compared to other types of strategies. In total, 45 students participated in these studies. The results are mixed. The first study, with a quasi-experimental design carried out by Jongmans et al. (2003), did not show gains in writing fluency in the intervention group (condition 1 in Table 1) in relation to the control group (condition 2 in Table 1). On the other hand, it was found in the study by Zumbrunn and Bruning (2012), implementing a self-regulated strategy combined with self-regulation procedures such as monitoring and goal setting, showed that participants wrote stories that contained more essential components, were longer, and had better quality after the treatment. However, the information provided did not allow for the calculation of the ES. A third study, by Kasper-Ferguson and Moxley (2002), reported the results of student graphing of writing fluency (monitoring their writing fluency) after brief freewriting periods. The primary goal was to increase writing fluency. The writing rates from all students improved over the course of the school year. These improvements in writing rate occurred without evidence of ceiling effects over a year’s time. Finally, Geisler et al.’s (2009) study examined the effects of self-counting and study use of synonym lists on the number of total words written by high-achieving students. All five students increased the amount of writing they produced in the intervention phase compared to baseline results.

#### Performance Feedback

In order to determine if performance feedback showed improvement in writing fluency, a total of seven studies were analyzed; 796 students participated in these studies. The amount of instruction varied from eight weeks to one academic year. Of these studies, six had a True-QES design (Roth and Guinee, 2011; Hier and Eckert, 2014, 2016; Truckenmiller et al., 2014; Alitto et al., 2016; Koenig et al., 2016) and showed a combined ES of 0.36 [95% CI (0.06, 0.66)]. The significant Q and I^2^ value of 70.34 indicated that the effects may imply considerable heterogeneity. The Fail-safe N indicated that 58 missing studies would be needed to reverse this finding. One non-experimental study reported gains in writing fluency associated with the use of performance feedback (Heskial and Wamba, 2013), highlighting that kindergarten students benefit from engagement in dialogue with the teacher, who read the feedback to them in order to support their development of a sense of story.

## Discussion

In comparison with other academic areas such as reading, little attention has been directed to preventing writing difficulties. The findings from the previous reviewed studies, however, indicate that handwriting treatments early in kindergarten and the primary grades may be a critical factor to improve and to prevent writing fluency difficulties, for full range students as well as for students with handwriting difficulties in producing letters fluently and automatically.

Similar to treatments for improving reading fluency, different theoretical and empirical frameworks have been found in the reviewed studies to enhance writing fluency; these can be grouped into two broad categories: skill-based and performance-based interventions (Chafouleas et al., 2004). Skill-based strategies involve the use of antecedent teaching procedures such as handwriting, teaching transcription skills, spelling, and sensory-motor handwriting interventions. In contrast, performance-based strategies implicate the manipulation of consequences for fluent writing. These strategies typically incorporate an element of reinforcement in the form of programmed contingencies, self-regulated strategies, goal setting, and performance feedback. In some cases, skill-based as well as performance-based interventions to improve writing fluency incorporate peer-assisted strategies (Puranik et al., 2017, 2018). Skill- and performance-based instructional strategies can be used in isolation or combined both within and across categories (Chafouleas et al., 2004).

As expected, we found that skill-based as well as performance-based writing interventions enhance writing fluency. The findings from this meta-analysis and systematic review have important theoretical implications for writing fluency development among initial writers (kindergarten to 6th grade). These findings apply to students with and without handwriting difficulties in kindergarten through 6th grade. While different types of handwriting instruction improved fluency, there was considerable variability in the magnitude of the effects.

In the first place, we analyzed whether any type of handwriting instruction produced greater effects in writing fluency than no instruction or instruction unrelated to handwriting, such as phonological awareness or keyboarding. We located 13 studies including students representing a full range of handwriting skills. They included students in kindergarten to through 5th grade. Although all the intervention conditions involved a type of handwriting instruction, the specific type of intervention and tasks of focus varied greatly across the 13 studies. Three of them focused on performance-based interventions (e.g., performance feedback or goal setting). Five of them focusing on skill-based interventions (e.g., writing letters, words, and sentences fluently and accurately; one of the studies used sensory-motor handwriting practices). Finally, five of them were multicomponent programs using combined skill- and performance-based interventions. The result show that handwriting instruction had a statistically significant impact on improving writing fluency.

Secondly, we categorized studies by the intervention type tested in their experimental conditions: handwriting/transcription skills, handwriting combined treatments, multi-component programs, sensory-motor handwriting interventions, self-regulated strategy, and performance feedback (see Table 1). The handwriting instruction presented ranged from relatively short and focused interventions (e.g., copying letters during a few sessions) to longer and more comprehensive handwriting programs (e.g., multi-component instruction spanning several months). As noted earlier, we calculated only a summary statistic (ES) for treatments that included at least three studies. We recognize, however, that small sample sizes are less reliable and must be interpreted more cautiously than a summary statistic based on a larger number of studies.

One skill-based strategy that was shown to be effective in isolation or combined is handwriting/transcription intervention, especially when it is aimed at writing automatically and fluently (Graham et al., 2000, 2018; Alves et al., 2016; Limpo and Alves, 2018; Limpo et al., 2018). The reviewed research indicated robust significant differences in writing fluency for primary grade students assigned to a handwriting treatment alone (see Tables 1, 3) or combined (see Table 1). However, in the handwriting/transcription intervention group, two out of five studies (Mackay et al., 2010; Howe et al., 2013) did find negative effects in handwriting fluency after treatment. Nonetheless, Howe et al. (2013) reported limitations in the test used to score writing fluency. The authors claimed that the test used did not accurately assess differences in speed between students. Moreover, the study by Mackay et al. (2010) did not explicitly train handwriting fluency and although students gained in writing legibility, but they obtained lower scores in writing speed after treatment. In the case of the combined handwriting intervention, Hurschler Lichtsteiner et al. (2018) reported that, as handwriting automaticity was already high at the beginning of the study, the intervention was not able to improve it further.

Due to the important role of transcription skills, handwriting must be trained not only to be as legible as possible, but also to become more and more fluent (Hurschler Lichtsteiner et al., 2018). The results of the present review show that when handwriting programs solely teach the components of legibility (Mackay et al., 2010) or spelling (Alves et al., 2016; Hurschler Lichtsteiner et al., 2018), writing fluency does not improve. The same conclusion could be applied to sensory-motor handwriting treatments not centered on training writing fluency practices. In general terms, these programs improve the readability of handwriting and other handwriting measures, such as letter formation and spatial organization, but not writing speed (Weintraub et al., 2009; Salls et al., 2013). In the category of sensory-motor handwriting treatment, we found one exception, i.e. the study by Roberts et al. (2010). They reported a significant increase in handwriting speed; however, the authors mentioned that this gain in writing speed was clinically insignificant compared to other improvements the students obtained after the treatment. However, we have to be aware that there were not enough data to calculate the impact of sensory-motor handwriting intervention and to make informed conclusions.

In the reviewed research, we also found additional practices, involving highly structured and explicit multi-component writing instruction programs such as Peer-Assisted Writing Strategies (PAWS) (Puranik et al., 2017, 2018), Write Star (Case-Smith et al., 2011, 2012, 2014a,b), and I Can! (Van Waelvelde et al., 2017). These programs include skill-based instructional strategies and performance-based strategies used in combination. The PAWS program applies two theoretical and empirical frameworks for beginning writers. The first focus of the program is on teaching transcription skills, i.e., handwriting and spelling. The second focus is on writing as a mode of social action that allows a child to learn from interaction with a more knowledgeable other. The teacher models the lessons, and the feedback processes and learning occurs during interactions among students and teacher and between students acting as coaches and writers (Puranik et al., 2017, 2018). The Write Star program (Case-Smith et al., 2011, 2012, 2014a,b) includes six core elements: (1) a co-teaching model of two teachers and an occupational therapist, (2) the teacher and therapist model letter formation, (3) the students copy from the model and engage in repeated practice, (4) the students work in groups of 6-7 that rotate through sessions, (5) the teachers and therapist provide frequent feedback, encouraging self-evaluation, and praising the students’ efforts, and (6) the teachers and therapist monitor and assess students’ performance, combining skill- and performance-based writing intervention strategies. Regarding the I Can! program, it should be mentioned that this is a remedial handwriting program with a focus on self-regulated learning and applying motor learning principles combined with handwriting practices and a behavioral approach (Van Waelvelde et al., 2017). All these multicomponent programs are good examples of using a multi-element design approach. ES and meta-analysis results of the present study suggested that multi-component programs, i.e., a combination of skill-based and performance-based intervention incorporating peer-assisted learning strategies, could be an adequate approach to develop writing fluency in early writers.

The effectiveness of the self-regulated strategy to improve writing fluency is mixed. Jongmans et al. (2003) tested a handwriting self-instruction method centered on how to form letter shapes and sensory-motor-learning principles. They found that children in the treatment group did not improve in writing fluency. The ES of the rest of the studies in this category could not be calculated because data were not available. Geisler et al. (2009) examined the effects of students’ self-counting of words on the total words written, showing that all five students increased the amount of writing they produced. Zumbrunn and Bruning (2012) implemented a self-regulated strategy development model of instruction involving teaching students’ strategies for planning and organizing their writing, combined with self-regulation procedures, such as monitoring and goal setting. The authors describe that, after treatment, participants wrote stories that contained more essential components, were longer, and of better quality. Kasper-Ferguson and Moxley (2002) report on the results of student graphing and monitoring their writing fluency. Their primary goal was to increase writing fluency, and they found that the writing rates of all students improved over the course of the school year. It is worth mentioning in this category the study by Limpo and Alves (2018) that examined the effectiveness of combining self-regulation and handwriting training with a self-regulation only intervention. The results showed that the self-regulation only intervention was particularly uneffective at increasing writing fluency.

Additionally, performance feedback (PF) was shown to be effective in a number of academic domains, including writing, and it has been implemented with students at all ages (Eckert et al., 2009). Research into the effectiveness of feedback on behavior has a long history in Psychology, since feedback has been identified as a mechanism that leads to a change in behavior, because the student compares that feedback to some standard of performance and feels motivated either to reduce the discrepancy or to surpass the standard (Bandura, 1969).

The results from several reviewed randomized control trials (Hier and Eckert, 2014, 2016; Truckenmiller et al., 2014; Alitto et al., 2016; Koenig et al., 2016) have indicated that performance feedback is a simple-to-implement, time-efficient method to improve writing fluency in elementary-age students. Students engaged on a brief Curriculum Based Measurement in Written Expression (CBM-WE) probe delivered once per week over the course of several weeks. These probes consist of giving the students a prompt for writing a story. These prompts were read aloud to students as well as printed on their response sheets. Students were given one minute to plan their stories and three minutes to write their compositions. Performance feedback conditions included students receiving feedback regarding their performance on the CBM-WE probes from the previous session. In comparison to students who engaged in weekly writing practice without feedback, those who received feedback about their writing fluency demonstrated significantly greater growth in writing fluency over the course of the intervention. Further to the positive impact of the performance feedback intervention on students’ writing fluency, teachers rated the PF intervention as acceptable on measures of social validity (Hier and Eckert, 2016) and as positively affecting students’ self-efficacy, a variable that contributes to overall writing competence (Hier and Mahony, 2018). However, the effect of intervention maintenance was limited (Hier and Eckert, 2014; Hier and Mahony, 2018). These findings suggest that, in isolation, performance feedback may produce short-term desired effects on students’ writing fluency growth, but explicit programming of generality may be required to produce long-term achievement gains. Although the performance feedback intervention leads to improvements in students’ performance in writing fluency, research by Alitto et al. (2016) found that the combination of performance feedback, provided by teachers or peers, combined with goal setting, leads to better performance than either component alone. In contrast, a study by Koenig et al. (2016) showed that providing students with an additional goal-setting component did not improve students’ writing fluency more than performance feedback alone.

Additionally, a study by Roth and Guinee (2011) showed that children made gains in sentence fluency after an interactive writing intervention in which the teacher provided powerful demonstrations of writing and delivered the clearest examples of instruction in response to the learners’ needs; moreover, children had the opportunity to work together to solve problems. In the same vein, a study by Heskial and Wamba (2013) highlighted that kindergarten students benefit from engagement in a dialogue with their teacher, who read the feedback to them in order to support their development of a sense of story. Kindergarten students responded to teacher feedback in different ways, but the feedback contributed to an increase in writing fluency that was consistent and evident in the analyzed studies.

## Limitations and Conclusion

Interpretation of the findings for specific instructional techniques should be considered by the fact that the number of studies testing each practice was small. Additional research is needed to more fully test the effectiveness of these interventions.

We adopted a liberal, exploratory approach. Although this allowed us to optimize the findings from the available research, it also meant many of our analyses were underpowered. However, we indicated in each analyzed handwriting intervention category how much confidence can be placed in our results, based on the indicators of the quality of the included research (see Table 4).

The studies reviewed here allowed us to establish that handwriting treatments early in kindergarten and the primary grades compared to non-treatment enhance writing fluency for developing writers and may be a critical factor to improve and to prevent writing fluency difficulties for full range students as well as for students with learning disabilities. This finding provides support for the significance of developing automaticity of the transcription component of the Simple View of Writing for initial writers (Berninger et al., 2002).

The present study explored the nature of writing fluency interventions in languages considered to have a non-transparent orthography. Out of the seventeen studies included in the performed meta-analyses, fourteen were in English, one in French (Van Waelvelde et al., 2017), one in Portuguese (Limpo and Alves, 2018) and another one in Hebrew (Weintraub et al., 2009) (see Table 1).

The present review also provides some insight into the effectiveness of specific methods for teaching handwriting fluency. Handwriting/transcription instruction is an effective to method to improve writing fluency, especially when it is aimed at promoting writing automaticity and students receive explicit instruction in writing letters, words, and sentences fluently and accurately. The results also show that multi-component handwriting programs may boost and prevent handwriting fluency difficulties in kindergarten and first-grade children. Finally, performance feedback interventions with writing practice based on CBM-WE probes demonstrated significant effects on writing fluency improvements after treatment. Other handwriting interventions, such as sensory-motor handwriting or self-regulation strategies, do not seem to have a significant impact on improving writing fluency. However, the current results show that planning explicit methods of writing intervention has an impact on improving writing fluency in early writers and that writing fluency intervention can be a means of preventing writing difficulties and a motivation to improve writing skills throughout schooling and for use in everyday life.

## Data Availability Statement

The raw data supporting the conclusions of this article will be made available by the authors, without undue reservation.

## Author Contributions

CL-E contributed to conception and design of the study and wrote the first draft of the manuscript. RP-L organized the database. JM-B performed the statistical analysis. CL-E, RP-L and JM-B wrote sections of the manuscript. All authors contributed to manuscript revision, read, and approved the submitted version.

## Conflict of Interest

The authors declare that the research was conducted in the absence of any commercial or financial relationships that could be construed as a potential conflict of interest.

## Publisher’s Note

All claims expressed in this article are solely those of the authors and do not necessarily represent those of their affiliated organizations, or those of the publisher, the editors and the reviewers. Any product that may be evaluated in this article, or claim that may be made by its manufacturer, is not guaranteed or endorsed by the publisher.

## References

[B1] Alitto J. Malecki C. K. Coyle S. Santuzzi A. (2016). Examining the effects of adult and peer mediated goal setting and feedback interventions for writing: two studies. *J. Sch. Psychol.* 56 89–109. 10.1016/j.jsp.2016.03.002 27268571

[B2] Alves R. A. Limpo T. (2015). Progress in written language bursts, pauses, transcription, and written composition across schooling. *Sci. Stud. Read.* 19 374–391.

[B3] Alves R. Limpo T. Fidalgo R. Carvalhais L. Pereira L. Castro S. (2016). The impact of promoting transcription on early text production: effects on bursts and pauses, levels of written language, and writing performance. *J. Educ. Psychol.* 108 665–679. 10.1037/edu0000089

[B4] Bandura A. (1969). *Principles of Behavior Modification.* New York, NY: Holt, Rinehart, and Winston.

[B5] Bara F. Bonneton-Botté N. (2017). Learning letters with the whole body: visuomotor versus visual teaching in kindergarten. *Percept. Mot. Skills* 125 190–207. 29161949 10.1177/0031512517742284

[B6] Bazerman C. Graham S. Applebee A. N. Matsuda P. K. Berninger V. W. Murphy S. (2017). Taking the long view on writing development. *Res. Teach. English* 51 351–360.

[B7] Berninger V. W. Fuller F. Whitaker D. (1996). A process model of writing development across the life span. *Educ. Psychol. Rev.* 8 193–218. 10.1007/BF01464073

[B8] Berninger V. W. Rijlaarsdam G. Fayol M. (2012). “Mapping research questions about translation to methods, measures, and models,” in *Translation of Thought to Written Text While Composing. Advancing Theory, Knowledge, Research Methods, Tools, and Applications*, eds Fayol M. Alamargot D. Berninger V. (East Sussex: Psychology Press), 27–70.

[B9] Berninger V. W. Rutberg J. E. Abbott R. D. Garcia N. Anderson-Youngstrom M. Brooks A. (2006). Tier 1 and Tier 2 early intervention for handwriting and composing. *J. Sch. Psychol.* 44 3–30. 10.1016/j.jsp.2005.12.003

[B10] Berninger V. W. Vaughan K. Abbott R. D. Begay K. Coleman K. B. Curtin G. (2002). Teaching spelling and composition alone and together: implications for the simple view of writing. *J. Educ. Psychol.* 94:291. 10.1037/0022-0663.94.2.291

[B11] Berninger V. Yates C. Cartwright A. Rutberg J. Remy E. Abbott R. (1992). Lower-level developmental skills in beginning writing. *Read. Writ. Interdiscip. J.* 4 257–280. 10.1007/BF01027151

[B12] Borenstein M. Hedges L. V. Higgins J. P. T. Rothstein H. R. (2009). *Introduction to Meta–Analysis.* Chichester: Wiley.

[B13] Case-Smith J. Holland T. Bishop B. (2011). Effectiveness of an integrated handwriting program for first-grade students: a pilot study. *Am. J. Occup. Ther.* 65 670–678. 10.5014/ajot.2011.000984 22214111

[B15] Case-Smith J. Weaver L. Holland T. (2014b). Effects of a classroom-embedded occupational therapist-teacher handwriting program for first-grade students. *Am. J. Occup. Ther.* 68 690–698. 10.5014/ajot.2014.011585 25397764 PMC4234841

[B14] Case-Smith J. Holland T. White S. (2014a). Effectiveness of a co-taught handwriting program for first grade students. *Phys. Occup. Ther. Pediatr.* 34 30–43. 10.3109/01942638.2013.783898 23607325

[B16] Case-Smith J. Holland T. Lane A. White S. (2012). Effect of a coteaching handwriting program for first graders: one group pretest-posttest design. *Am. J. Occup. Ther.* 66 396–405. 10.5014/ajot.2012.004333 22742687

[B17] Chafouleas S. M. Martens B. K. Dobson R. L. Weinstein K. S. Gardner K. B. (2004). Fluent reading as the improvement of stimulus control: additive effects of performance-based interventions to repeated reading on students’ reading and error rates. *J. Behav. Educ.* 13 67–81. 10.1023/b:jobe.0000023656.45233.6f

[B18] Cohen J. (1988). *Statistical Power Analysis for the Behavioral Sciences*, 2nd Edn. Hillsdale, NJ: Lawrence Erlbaum Associates, Publishers.

[B19] Cooper H. Hedges L. V. Valentine J. C. (2019). *The Handbook of Research Synthesis and Meta-Analysis*, 3rd Edn. New York, NY: Russell Sage Foundation.

[B20] Eckert T. L. Codding R. M. Truckenmiller A. J. Rheinheimer J. J. (2009). “Improving children’s fluency in reading, mathematics, spelling, and writing: a review of evidence-based academic interventions,” in *Behavioral Interventions in School. Evidence-Based Positive Strategies*, eds Akin-Little A. Little S. G. Bray M. A. Kehle T. J. (Washington, DC: American Psychological Association), 111–124. 10.1037/11886-007

[B21] Edwards L. (2003). Writing instruction in kindergarten: examining and emerging area of research for children with writing and reading difficulties. *J. Learn. Disabil.* 36 136–148. 10.1177/002221940303600206 15493429

[B22] Egger M. Smith G. D. Schneider M. Minder C. (1997). Bias in meta-analysis detected by a simple, graphical test. *Br. Med. J.* 315 629–634. 10.1136/bmj.315.7109.629 9310563 PMC2127453

[B23] Finlayson K. McCrudden T. (2019). Teacher-implemented self-regulated strategy development for story writing with 6-years-olds in a whole-class in New Zealand. *J. Res. Child. Educ.* 33 307–322. 10.1080/02568543.2019.1568328

[B24] Geisler J. Hessler T. Gardner R. Lovelace T. (2009). Differentiated writing interventions for high-achieving urban African American elementary students. *J. Adv. Acad.* 20 214–247. 10.1177/1932202X0902000202

[B25] Gersten R. Fuchs L. Compton D. Coyne M. Greenwood C. Innocenti M. S. (2005). Quality indicators for group experimental and quasi-experimental research in special education. *Exceptional Child.* 71 149–164. 10.1177/001440290507100202

[B26] Gilbert J. Graham S. (2010). Teaching writing to elementary students in Grades 4-6: a National Survey. *Element. Sch. J.* 110 494–518. 10.1086/651193

[B27] Graham S. Harris K. R. (2018). “Evidence-based writing practices: a meta-analysis of existing meta-analyses,” in *Design Principles for Teaching Effective Writing. Theoretical and Empirical Grounded Principles*, Vol. 34 eds Fidalgo R. Harris K. R. Braaksma M. (Leiden: Brill), 13–37. 10.1163/9789004270480_003

[B28] Graham S. Perin D. (2007). *Writing Next: Effective strategies to Improve Writing of Adolescents in Middle and High Schools - A report to Carnegie Corporation of New York. Alliance for Excellent Education.* Leiden: Koninklijke Brill NV.

[B29] Graham S. Sandmel K. (2011). The process writing approach: a meta-analysis. *J. Educ. Res.* 104 396–407. 10.1080/00220671.2010.488703

[B30] Graham S. Berninger V. W. Abbott R. D. Abbott S. P. Whitaker D. (1997). Role of mechanics in composing of elementary school students: a new methodological approach. *J. Educ. Res.* 89 170–182. 10.1037/0022-0663.89.1.170

[B31] Graham S. Harris K. R. Adkins M. (2018). The impact of supplemental handwriting and spelling instruction with first grade students who do not acquire transcription skills as rapidly as peers: a randomized control trial. *Read. Writ.* 31 1273–1294.

[B32] Graham S. Harris K. R. Fink B. (2000). Is handwriting causally related to learning to write? Treatment of handwriting problems in beginning writers. *J. Educ. Psychol.* 92 620–633. 10.1037/0022-0663.92.4.620

[B33] Graham S. Harris K. R. Santangelo T. (2015). Research-based writing practices and the common core. Meta-analysis and Meta-synthesis. *Element. Sch. J.* 115 498–522. 10.1086/681964

[B34] Graham S. McKeown D. Kiuhara S. Harris K. R. (2012). A meta-analysis of writing instruction for students in the elementary grades. *J. Educ. Psychol.* 104 879–896. 10.1037/a0029185

[B35] Hansen B. D. Wills H. P. (2014). The effects of goal setting, contingent reward, and instruction on writing skills. *J. Appl. Behav. Anal.* 47 171–175. 10.1002/jaba.92 24114533

[B36] Hayes J. R. (2012). “Evidence from language bursts, revision, and transcription for translation and its relation to other writing processes,” in *Translation of Thought to Written Text While Composing. Advancing Theory, Knowledge, Research Methods, Tools, and Applications*, eds Fayol M. Alamargot D. Berninger V. W. (East Sussex: Psychology Press), 15–26.

[B37] Hayes J. R. Flower L. (1980). “Identifying the organization of the writing process,” in *Cognitive Processes in Writing*, eds Gregg W. Steinberg E. R. (Mahwah, NJ: Lawrence Erlbaum), 3–30.

[B38] Hedges L. V. (1981). Distribution theory for Glass’s estimator of effect size and related estimators. *J. Educ. Stat.* 6 107–128. 10.2307/1164588

[B39] Heskial J. Wamba N. G. (2013). Lifting kindergarteners’ writing to meet the common core learning standards: a collaborative inquiry. *Can. J. Act. Res.* 14 51–64.

[B40] Hier B. O. Eckert T. L. (2014). Evaluating elementary-aged students’ abilities to generalize and maintain fluency gains of a performance feedback writing intervention. *Sch. Psychol. Q.* 29 488–502. 10.1037/spq0000040 24708279

[B41] Hier B. O. Eckert T. L. (2016). Programming generality into a performance feedback writing intervention: a randomized controlled trial. *J. Sch. Psychol.* 56 111–131. 10.1016/j.jsp.2016.03.003 27268572

[B42] Hier B. O. Mahony K. E. (2018). The contribution of mastery experiences, performance feedback, and task effort to elementary-aged students’ self-efficacy in writing. *Sch. Psychol. Q.* 33 408–418. 10.1037/spq0000226 28857589

[B43] Higgins J. P. T. Green S. (eds) (2011). *Cochrane Handbook for Systematic Reviews of Interventions (Version 5.1.0).* Available online at: https://training.cochrane.org/handbook/current

[B44] Howe T.-H. Roston K. L. Sheu C.-F. Hinojosa J. (2013). Assessing handwriting intervention effectiveness in elementary school students: a two-group controlled study. *Am. J. Occup. Ther.* 67 19–27. 10.5014/ajot.2013.005470 23245779

[B45] Hoy M. M. P. Egan M. Y. Feder K. P. (2011). A systematic review of interventions to improve handwriting. *Can. J. Occup. Ther.* 78 13–25. 10.2182/cjot.2011.78.1.3 21395194

[B46] Hurschler Lichtsteiner S. Wicki W. Falmann P. (2018). Impact of handwriting training on fluency, spelling and text quality among third graders. *Read. Writ. Interdiscip. J.* 31 1295–1318. 10.1007/s11145-018-9825-x 29875546 PMC5966469

[B47] Jensen L. A. Allen M. N. (1996). Meta-synthesis of qualitative findings. *Qual. Health Res.* 6 553–560. 10.1177/104973239600600407

[B48] Jones D. Christensen C. A. (1999). Relationship between automaticity in handwriting and students’ ability to generate written text. *J. Educ. Psychol.* 91 44–49. 10.1037/0022-0663.91.1.44

[B49] Jones D. Christensen C. A. (2012). “Impact of teacher professional development in handwriting on improved student learning outcomes in writing quality,” in *Translation of Thought to Written Text While Composing. Advancing Theory, Knowledge, Research Methods, Tools, and Applications*, eds Fayol M. Alamargot D. Berninger V. (East Sussex: Psychology Press), 213–228.

[B50] Jongmans M. J. Linthorst-Bakker E. Westenberg Y. Smits-Engelsman B. C. (2003). Use of a task-oriented self-instruction method to support children in primary school with poor handwriting quality and speed. *Hum. Mov. Sci.* 22 549–566. 10.1016/j.humov.2003.09.009 14624833

[B51] Kasper-Ferguson S. Moxley R. A. (2002). Developing a writing package with student graphing of fluency. *Educ. Treat. Child.* 25 249–267.

[B52] Kim Y.-S. G. Gatlin B. Al Otaiba S. Wanzek J. (2018). Theorization and an empirical investigation of the component-based and developmental text writing fluency construct. *Grantee Submission* 51 320–335. 10.1177/0022219417712016 28597728 PMC5711641

[B53] Kim Y.-S. Al Otaiba S. Puranik C. Folsom J. S. Greulich L. Wagner R. K. (2011). Componential skills of beginning writing: an exploratory study. *Learn. Individ. Differ.* 21 517–525. 10.1016/j.lindif.2011.06.004 22267897 PMC3261783

[B54] Koenig E. A. Eckert T. L. Hier B. O. (2016). Using performance feedback and goal setting to improve elementary students’ writing fluency: a randomized controlled trial. *Sch. Psychol. Rev.* 45 275–295. 10.17105/spr45-3.275-295

[B55] Koster M. Tribushinina E. De Jong P. F. Van den Bergh B. (2015). Teaching children to write: a meta-analysis of writing intervention research. *J. Writ. Res.* 7 299–324.

[B56] LaBerge D. Samuels S. J. (1974). Toward a theory of automatic information processing in reading. *Cogn. Psychol.* 6 293–323. 10.1016/0010-0285(74)90015-2

[B57] Limpo T. Alves R. A. (2018). Tailoring multicomponent writing interventions: effects of coupling self-regulation and transcription training. *J. Learn. Disabil.* 51 381–398. 10.1177/0022219417708170 28489968

[B58] Limpo T. Parente N. Alves R. A. (2018). Promoting handwriting fluency in fifth graders with slow handwriting: a single-subject design study. *Read. Writ. Interdiscip. J.* 31 1343–1366. 10.1007/s11145-017-9814-5

[B59] Mackay N. McCluskey A. Mayes R. (2010). The Log Handwriting Program improved children’s writing legibility: a pretest-posttest study. *Am. J. Occup. Ther.* 64 30–36. 10.5014/ajot.64.1.30 20131562

[B60] Martens B. K. Edward J. D. Begeny J. C. VanDerHeyden A. (2011). “Behavioral approaches to education,” in *Handbook of Applied Behavior Analysis*, eds Fisher W. W. Piazza H. S. Roane H. S. (New York, NY: The Guilford Press), 385–401. 10.1016/s0003-9993(95)80029-8

[B61] National Center for Education Statistics (2012). *The Nation’s Report Card: Writing 2011 (NCES 2012-470).* Washington, DC: Institute of Education Sciences, U.S. Department of Education.

[B62] Olive T. Kellogg R. T. (2002). Concurrent activation of high- and low-level production processes in written composition. *Mem. Cogn.* 30 594–600. 10.3758/BF03194960 12184560

[B63] Page M. McKenzie J. Bossuyt P. Boutron I. Hoffmann T. Mulrow C. (2021). The Prisma 2020 statement: an updated guideline for reporting systematic reviews. *BMJ* 372:n71. 10.1136/bmj.n71 33782057 PMC8005924

[B64] Peverly S. T. (2006). The importance of handwriting speed in adult writing. *Dev. Neuropsychol.* 29 197–216. 10.1207/s15326942dn2901_10 16390294

[B65] Pressley M. Graham S. Harris K. (2006). The state of educational intervention research as viewed through the lens of literacy intervention. *Br. J. Educ. Psychol.* 76 1–19. 10.1348/000709905X66035 16573976

[B66] Puranik C. S. Petscher Y. Al Otaiba S. Lemons C. J. (2018). Improving kindergarten students’ writing outcomes using peer-assisted strategies. *Element. Sch. J.* 118 680–710. 10.1086/697432

[B67] Puranik C. Patchan M. Lemons C. J. Al Otaiba S. (2017). Using peer assisted strategies to teach early writing: results of a pilot study to examine feasibility and promise. *Read. Writ.* 30 25–50. 10.1007/s11145-016-9661-9

[B68] Reisman J. (1999). *Minnesota Handwriting Assessment.* Harcourt: Harcourt Assessment.

[B69] Roberts G. I. Siever J. E. Mair J. A. (2010). Effects of a kinesthetic cursive handwriting intervention for grade 4-6 students. *Am. J. Occup. Ther.* 64 745–755. 10.5014/ajot.2010.08128 21073105

[B70] Rogers L. Graham S. (2008). A meta-analysis of single subject design writing intervention research. *J. Educ. Psychol.* 100 879–906. 10.1037/0022-0663.100.4.879

[B71] Roth K. Guinee K. (2011). Ten minutes a day: the impact of Interactive Writing instruction on first graders’ independent writing. *J. Early Child. Lit.* 11 331–361. 10.1177/1468798411409300

[B72] Salls J. Benson J. Hansen M. Cole K. Pielielek A. (2013). A comparison of the handwriting without tears program and Peterson directed handwriting program on handwriting performance in typically developing first grade students. *J. Occup. Ther. Sch. Early Interv.* 6 131–142. 10.1080/19411243.2013.810958

[B73] Santangelo T. Graham S. (2013). “Meta-analyses of handwriting and spelling instruction,” in *Paper Presented at the Pacific Coast Research Conference*, (Mission Beach, CA). 10.1044/2018_LSHSS-DYSLC-18-0024

[B74] Santangelo T. Graham S. (2016). A comprehensive meta-analysis of handwriting instruction. *Educ. Psychol. Rev.* 28 225–265. 10.1007/s10648-015-9335-1

[B75] Suurmond R. van Rhee H. Hak T. (2017). Introduction, comparison and validation of Meta-Essentials: a free and simple tool for meta-analysis. *Res. Synth. Methods* 8 537–553. 10.1002/jrsm.1260 28801932 PMC5725669

[B76] Truckenmiller A. J. Eckert T. L. Codding R. S. Petscher Y. (2014). Evaluating the impact of feedback on elementary aged students’ fluency growth in written expression: a randomized controlled trial. *J. Sch. Psychol.* 52 531–548. 10.1016/j.jsp.2014.09.001 25432270 PMC5046133

[B77] Van Waelvelde H. De Roubaix A. Steppe L. Troubleyn E. De Mey B. Dewitte G. (2017). Effectiveness of a self-regulated remedial program for handwriting difficulties. *Scand. J. Occup. Ther.* 24 311–319. 10.1080/11038128.2017.1282041 28276960

[B78] Vander Hart N. Fitzpatick P. Cortesa C. (2010). In depth analysis of handwriting curriculum and instruction in four kindergarten classrooms. *Read. Writ.* 23 673–699. 10.1007/s11145-009-9178-6

[B79] Weintraub N. Yinon M. Hirsch I. Parush S. (2009). Effectiveness of sensorimotor and task-oriented handwriting intervention in elementary school-aged students with handwriting difficulties. *OTJR Occup. Particip. Health* 29 125–134. 10.3928/15394492-20090611-05

[B80] Wicki W. Hurschler Lichtsteiner S. Saxer Geiger A. Müller M. (2014). Handwriting fluency in children: impact and correlates. *Swiss J. Psychol.* 73 87–96. 10.1024/1421-0185/a000127

[B81] Wolf M. Katzir-Cohen T. (2001). Reading fluency and its intervention. *Sci. Stud. Read.* 5 211–239. 10.1207/S1532799XSSR0503_2

[B82] Zumbrunn S. Bruning R. (2012). Improving the writing and knowledge of emergent writers: the effects of self-regulated strategy development. *Read. Writ.* 26 91–110. 10.1007/s11145-012-9384-5

